# Editorial Notes

**Published:** 1930

**Authors:** 


### Editorial Notes

**Granuloma Laryngis; a Collective Investigation.**

MR. E. WATSON-WILLIAMS has been invited to collate cases of (? papillary) granuloma of the larynx, or of a similar nature, with special reference to occurrence after exposure to mustard or other irritant gas, for presentation at the Summer Meeting of the Laryngological Section of the Royal Society of Medicine. The points of note appear to be :—

1. Signs and symptoms, clinical appearances.
2. Date of exposure to gas, nature of latter.
3. Latent period, if any.
4. Operative measures ; exact site of granuloma ; *pathological report.*
5. Exclusion of syphilis, tubercle : blood or sputum examination ?
6. Subsequent history.

If any of our readers have met with cases of this hitherto little-recorded condition, Mr. Watson-Williams will be grateful to have reports forwarded to him at the Bristol Royal Infirmary. Illustrations, microscopic slides, and of course, when possible, personal presentation of cases or reports will be especially welcome. References to published cases or to occurrence apart from war service will be valued.

**Harvey Memorial Fund.**

AN appeal has been issued to the medical profession by an influential Committee whose Chairman is Sir John Rose Bradford, for funds to restore the tower of Hempstead Church in Essex, which collapsed in 1882. Here lie the remains of William Harvey, whose family was long associated with the church, a fourteenth-century building of considerable interest. The estimated cost of this restoration is £5,700, of which about £1,000 has been subscribed. It is hoped that a Bristol Committee may be formed for the collection of funds. We feel sure that the medical profession in the West of England will be glad of this opportunity to do honour to the memory of William Harvey, who did so much to establish the study of medicine on a basis of truthful and accurate observation.

**The Hunterian Oration, 1930 (76th).**

IF Harvey's achievements were on the grand scale, so also were John Hunter's, and Professor Hey Groves was well advised to entitle the 76th Hunterian Oration, which he delivered at the Royal College of Surgeons on February 14th, "Hero Worship in Surgery." He justified this title by reference both to Hunter's character and also to his achievements; dwelling in particular on his experiments in grafting. Professor Hey Groves is to be congratulated no less on the matter and the manner of his Oration than on the honour done to him by his election as Orator—the third time that this honour has fallen to a provincial surgeon, the others being George Murray Humphry of Cambridge and the present President of the Royal College of Surgeons, Lord Moynihan.

At the Hunterian Festival Dinner held the same evening at the College the Vice-Chancellor of the University of Bristol, Dr. Loveday, proposed the Orator's health, and expressed the pleasure that this recognition of Professor Hey Groves's great contributions to the advancement of surgery had given to his colleagues in Bristol.

